# A step toward understanding the mechanism of action of audit and feedback: a qualitative study of implementation strategies

**DOI:** 10.1186/s13012-021-01102-6

**Published:** 2021-04-01

**Authors:** Mellanie V. Springer, Anne E. Sales, Nishat Islam, A. Camille McBride, Zach Landis-Lewis, Michael Tupper, Casey L. Corches, Maria Cielito Robles, Lesli E. Skolarus

**Affiliations:** 1 Stroke Program, University of Michigan Medical School, 1500 E. Medical Center Drive, Ann Arbor, MI 48109 USA; 2 Department of Veteran Affairs Center for Clinical Management Research, Ann Arbor, MI USA; 3 Department of Learning Health Sciences, University of Michigan, Ann Arbor, MI USA; 4 School of Public Health, University of Michigan, Ann Arbor, MI USA; 5 University of Michigan Medical School, Ann Arbor, MI USA

**Keywords:** Audit and feedback, Implementation strategies, Mechanism of action, Causal pathway models, Stroke

## Abstract

**Background:**

Audit and feedback (A&F) is a widely used implementation strategy. Understanding mechanisms of action of A&F increases the likelihood that the strategy will lead to implementation of an evidence-based practice. We therefore sought to understand one hospital’s experience selecting and implementing an A&F intervention, to determine the implementation strategies that were used by staff and to specify the mechanism of action of those implementation strategies using causal pathway models, with the ultimate goal of improving acute stroke treatment practices.

**Methods:**

We selected an A&F strategy in a hospital, initially based on implementation determinants and staff consideration of their performance on acute stroke treatment measures. After 7 months of A&F, we conducted semi-structured interviews of hospital providers and administrative staff to understand how it contributed to implementing guideline-concordant acute stroke treatment (medication named tissue plasminogen activator). We coded the interviews to identify the implementation strategies that staff used following A&F and to assess their mechanisms of action.

**Results:**

We identified five implementation strategies that staff used following the feedback intervention. These included (1) creating folders containing the acute stroke treatment protocol for the emergency department, (2) educating providers about the protocol for acute stroke, (3) obtaining computed tomography imaging of stroke patients immediately upon emergency department arrival, (4) increasing access to acute stroke medical treatment in the emergency department, and (5) providing additional staff support for implementation of the protocol in the emergency department. We identified enablement, training, and environmental restructuring as mechanisms of action through which the implementation strategies acted to improve guideline-concordant and timely acute stroke treatment.

**Conclusions:**

A&F of a hospital’s acute stroke treatment practices generated additional implementation strategies that acted through various mechanisms of action. Future studies should focus on how initial implementation strategies can be amplified through internal mechanisms.

**Supplementary Information:**

The online version contains supplementary material available at 10.1186/s13012-021-01102-6.

Contributions to the literature

- Research studies describing the mechanism of action of audit and feedback, particularly action planning, are limited. Understanding how and why audit and feedback works can maximize its ability to produce implementation outcomes.
- We found that audit and feedback can lead to hospital staff generating and deploying additional implementation strategies, independent of researcher intervention.
- We identified internal and external factors that motivated the health care team to generate and deploy implementation strategies.
- Our findings can be used by other health care organizations to maximize the success of audit and feedback in increasing implementation of an evidence-based clinical practice.

## Background

Evidence-based interventions are sometimes slow to translate into routine clinical practice. The field of implementation science aims to decrease this time frame by using systematic approaches to improve uptake of evidence-based practices [1].

One component of a systematic approach based on tools from implementation science is implementation mapping, the process of selecting one or more implementation strategies based on identified barriers and facilitators, or determinants, to implementation success [2]. Methods for aligning a specific determinant to a specific implementation strategy are evolving. Improved understanding of how implementation strategies effect their outcomes will facilitate selection of the most appropriate implementation strategies for a particular determinant. Causal pathway models propose the mechanism of action by which implementation strategies effect their outcomes and may also specify factors that influence or are important for implementation [3]. Specification of implementation strategies leads to improved understanding of why a particular strategy is effective and in what circumstances it is effective, therefore contributing to the success of implementation efforts.

One implementation strategy that has been widely used in the clinical realm is audit and feedback (A&F). The focus in A&F implementation research has often been to evaluate whether it increases implementation of the evidence-based practice, rather than focus on the mechanism by which it leads to successful implementation. One theory about A&F specific to the implementation of healthcare-related behaviors is the Clinical Performance Feedback Intervention Theory (CP-FIT) which proposes that A&F acts through a feedback cycle which results in the optimization of individual patient care or modification of care delivery across the organization [4]. These patient- and organizational-level changes may lead to improved clinical performance. In essence, A&F can foster the development of additional implementation strategies. Specifying the mechanism of action of these further implementation strategies can facilitate their translation to similar clinical settings or refinement to enhance their impact.

The objective of the current research was to identify the implementation strategies that were developed by clinical teams, rather than by researchers, as a result of using A&F as an initial strategy to increase implementation of evidence-based acute stroke treatment. In this study, we investigate the implementation of tissue plasminogen activator (tPA) in the emergency department (ED) for acute stroke treatment. Patients treated with tPA are at least 30% more likely to have minimal or no disability at 3 months compared with patients who are not treated [5]. Since faster treatment is associated with less disability, treatment with tPA within 60 min of hospital arrival is a stroke quality metric recommended by the American Heart/Stroke Association [6]. Despite that tPA was approved by the United States Food and Drug Administration (FDA) for acute stroke treatment in 1996, national rates of tPA use are only 4.2% on average [7]. In this context, we used A&F as an implementation strategy to increase tPA use in the emergency department of a hospital in Flint, Michigan. We sought to identify implementation strategies that were developed by clinical teams as a result of A&F delivery and specify their mechanisms of action.

## Methods

### Setting

The study was conducted at a hospital in Flint, a predominantly African American city. Flint, Michigan has a tPA use rate well below the national average. We describe the systematic process used by the research team and the responses by clinicians and administrators within the hospital.

#### Determinant assessment

Guided by the Tailored Implementation for Chronic Disease (TICD) [8], the research team (for the remainder of this report, we refer to the research team as “we”) performed 15 interviews with Emergency Department providers [9]. In order to focus the interview guide into a subset of TICD domains, we conducted 2 workshops attended by vascular neurologists, ED physicians, ED and neurology nurses, stroke program directors, and the research team. The interview guide was then written by a stroke neurologist with experience in qualitative research (LS) and an implementation researcher (AS). Creation of the interview guide was an iterative process with collaboration between LS and AS until a consensus was reached (see Additional file 1). We selected interview participants by purposive sampling of providers caring for acute stroke patients in different clinical roles, settings, and leadership positions. Participants from the purposive sample suggested up to 3 potential participants who they thought could provide insight into tPA treatment. The stroke neurologist LS, who had no prior relationship with the participants and who does not practice at the hospital, conducted the interviews. Member checking and peer debriefing were performed to enhance trustworthiness of the data.

#### Prioritization and strategy selection

We presented these results to the 12-member acute stroke leadership team who formed a smaller task force to review the results in more depth and determine a response. The multidisciplinary task force was comprised of the lead stroke neurologist, stroke and neuro-ICU nursing leadership, and a hospital administrator who reviewed the identified determinants and discussed possible implementation strategies to address the locally identified gaps. The discussion was guided by the APEASE criteria: affordable, practical, effective, acceptable, safe, and equitable [10]. Each member was given a worksheet that listed implementation strategy options including feedback reports, restructured consent forms for stroke treatment, creation of an acute stroke team, nomination of a stroke champion, and improving documentation of the last time the stroke patient was known to be well (which is the time used as the basis for treatment decisions). Each member was asked to score each implementation strategy from 1—strongly disagree to 5—strongly agree on each of the APEASE criteria (see Additional file 2). The absolute scores were discussed and used to spur conversation regarding prioritization, but were not used as a quantitative assessment. The decision of which implementation strategy to move forward was ultimately determined by consensus.

### Creation of the feedback report

We worked with the task force to design the feedback report, which was then reviewed and suggestions for improvement were made by the acute stroke leadership team. We place our feedback report in the context of the CP-FIT theory [4], which formulates high-confidence hypotheses about the factors contributing to the success of feedback in implementation of evidence-based practices. Our feedback report incorporated the feedback variables defined by the CP-FIT theory, including a defined goal, appropriate method of data collection and analysis, and an effective feedback display and means of delivery. The goal of the A&F strategy was to optimize acute stroke care processes, as measured by a reduction in the time from ED arrival to tPA treatment. The broader goal was that optimizing acute stroke care processes would ultimately increase tPA use and increase hospital stroke awareness. These were recognized as achievable goals by the acute stroke leadership team. The feedback reports leveraged data from the electronic medical record that were already being collected for another purpose and could be produced internally, without the involvement of the research team. See Additional file 3 for description of the development of the A&F report.

Each feedback report stated the guidelines for tPA administration. They included tPA treatment rates and time-critical tPA delivery process measures aggregated over 3-month intervals. It was necessary to aggregate the data over the previous 3 months to provide stability to the data due to the low number of tPA cases and high variability in patient factors. Performance data about tPA treatment rates and tPA delivery process times were displayed in easy-to-read line and bar graphs with performance in the current quarter compared with previous quarters. Since tPA treatment is a team effort, performance was summarized across all ED provider types (physicians, nurses, medical techs). Feedback reports were emailed to all levels of ED providers and discussed monthly at ED nurse and medical technician huddles and ED physician meetings and the monthly acute stroke leadership team meeting (example of a feedback report is provided in Additional file 4). The research team generated feedback reports monthly over 10 months (October 2017 to July 2018) that were disseminated by the hospital. In December 2018, the A&F reports were transitioned to the hospital team.

### Qualitative interviews

Seven months after initiating A&F, we began to conduct semi-structured interviews of hospital providers to identify perceptions of A&F and the mechanism of action whereby A&F led to changes in delivery of tPA for acute stroke in the ED. We identified the implementation strategies other than A&F employed by hospital staff over the previous 6 months, which were initiated by individuals within the hospital, not by the research team.

The interview guides were grounded in implementation frameworks including the Capability, Opportunity, Motivation, Behavior (COM-B) model, Theory of Planned Behavior, and Theoretical Domain Framework (TDF) [11–13]. We chose these frameworks because these are theories of behavior change which can be applied to achieve our overarching goal of increasing implementation of acute stroke treatment practices. The interview guide was reviewed iteratively by the research team and modified accordingly for a total of 11 questions (see Additional file 5).

Interviews with 8 emergency department providers and 2 members of the administrative staff were conducted by a stroke neurologist (LS) between May and July of 2018, each lasting a mean of 20 min (± 5 min). The stroke neurologist LS has experience in semi-structured interviewing of research participants and has taken courses in qualitative research methods [9, 14, 15]. As the primary investigator of the research, LS had meetings with ED leadership to obtain buy-in for the research. LS had not previously partnered with ED leadership or staff on other research projects and was therefore considered to be a neutral interviewer having no relationship with the interviewees. Ten interviews achieved thematic saturation. Interviews took place at the hospital, typically during participant breaks in performing clinical care or administrative responsibilities. All interviews were recorded and transcribed. This study was approved by the University of Michigan’s Institutional Review Board. Participant consent was obtained. This study conforms to the Standards for Reporting Qualitative Research (see Additional file 6).

### Data analysis

#### Coding strategy

The purpose of qualitative analysis was to explore how A&F was used to improve acute stroke care. We used the framework method to analyze the data. Specifically, two research assistants (NI, CM) reviewed interview transcripts and used open coding, underlining text relevant to understanding how audit and feedback led to the implementation of acute stroke treatment. After independently coding the transcripts, the 2 research assistants met to develop a mutually agreed upon analytical framework using Michie’s Behavior Change Wheel [11] as a guide. If unable to reach consensus, LS resolved discrepancies. The data were charted with the implementation strategy as the heading, followed by quotes supporting that implementation strategy and the corresponding intervention function from Michie’s Behavior Change Wheel. We conceptualized each implementation strategy as an intervention. Thus, the intervention function from the COM-B system represented the mechanism by which an implementation strategy led to outcomes.

#### Overview of data analysis

Our coding of the interview transcripts enabled us to define implementation strategies and mechanisms of action for those strategies. Based on the identified mechanisms of action, we built causal pathway models that describe the pathway through which each implementation strategy achieved its distal outcome of tPA delivery. We then reviewed the data in the context of the CP-FIT theory as a framework to understand how the A&F reports may have led to the generation of implementation strategies by the hospital team. These steps in data analysis are further described below.

##### Step 1: implementation strategies

We followed the guidance of Proctor et al. [16] to define the implementation strategies. Specifically, we name, define, operationalize, and specify each implementation strategy in terms of the people carrying out the strategy (the actor), the intended target (action target), the dose, and the outcomes affected (Table 1). We verified the implementation strategies with the participants using synthesized member checking.

**Table 1:** Implementation strategies to improve acute stroke care measures

Implementation strategy	Definition of strategy	Specification of strategy				Outcomes affected
		Action	Actor	Action target	Dose (frequency)	
1. Detailed stroke care protocols in the ED	Provision of guidelines and instructions on how to implement stroke care readily available in ED	Develop and disseminate stroke care protocols in the ED	ED supervisor	ED providers	Ongoing	Standardize stroke care, improve quality of care, reduce time to acute stroke treatment, guideline-concordant tPA use
2. Stroke care education and tPA competency training	Education of staff on stroke protocols and guidelines and provision of interactive training on tPA administration	In-person group training on stroke protocols and tPA administrate-on	ED providers, nurse educators (doctors, nurses)	ED providers (doctors, nurses)	annual	Improve quality of care via increased awareness and familiarity with stroke protocols and self-efficacy with tPA administration, guideline-concordant treatment
3. Modification of the timing of CT brain imaging in acute stroke care	Taking the patient directly to the CT scanner upon hospital arrival	Routing patient to the CT scanner immediately on arrival	ED administrators	ED providers	Ongoing	Reduce time to acute stroke treatment (door-to-needle time)
4. Increased access to tPA in the ED	Storage of tPA in the ED	Storing tPA in the automated dispensing cabinets in the ED	Pharmacy	ED nurse	Ongoing	Reduce time to acute stroke treatment
5. Additional staff to support implementation of acute stroke care protocols	Supporting actions of ED staff during an acute stroke care protocol	Supporting behavior of ED staff during an acute stroke care protocol and providing assistance when needed	Stroke program manager, senior nurses, trauma nurse	ED staff involved in acute stroke care protocol	Ongoing	Increase fidelity of implementation of acute stroke care protocols to improve quality of care, timeliness of tPA treatment

*ED* emergency department, *tPA* tissue plasminogen activator, *CT* computed tomography

##### Step 2: causal pathway model

**Fig. 1:**
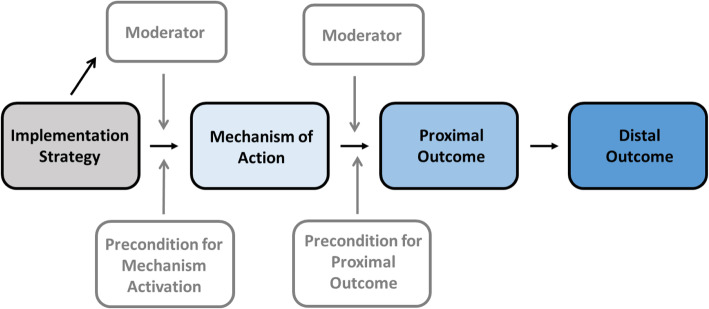
Causal pathway model

We defined the mechanism of action of each implementation strategy as the intervention function that emerged from qualitative coding of interviews. We then built causal pathway models by linking each implementation strategy through its mechanism of action to proximal and distal outcomes (Fig. 1 Causal pathway model). Proximal outcomes were defined as the measurable effect of implementation strategies based on our analysis of participant interviews. For distal outcomes, we hypothesized the effect of the proximal outcome on tPA administration. For preconditions and moderators, we hypothesized factors that might influence the mechanism of action and proximal outcome.

##### Step 3: how feedback reports led to implementation strategies

We used the CP-FIT theory to propose how the A&F reports may have led the hospital team to generate the additional implementation strategies.

## Results

### Outcome of determinant assessment

We identified that guideline factors, individual health professional factors, and patient factors were barriers to guideline concordant acute stroke thrombolysis [9]. The task force concluded that addressing guideline factors were most important, specifically that staff were not aware of the established standards for acute stroke treatment times, and individual health professional barriers, namely that feedback was given to leadership but was inconsistently given to the team that treated the patient and was not distributed consistently to direct care providers. A powerful persuasion point in the task force’s decision to use A&F reports was the specific request of their providers for feedback, that established guidelines for acute stroke treatment would be written on feedback reports and the belief that A&F was the first and most cost-effective step in comparison with any of the alternative strategies. Strategies that were not selected included restructuring of consent forms for acute stroke treatment to facilitate doctor–patient communication; however, tPA refusals were thought to be rare in their ED. Other strategies were to create a new acute stroke team, similar to the trauma team that was already in place, or a stroke champion to respond to the acute stroke, but these strategies were thought to be too costly. Finally, we considered improved documentation of when the patient was last known to be normal (assessing the time of stroke onset) to ensure potential patients were screened for eligibility for acute stroke treatment, which is a stroke treatment quality metric. This strategy was felt to have low effectiveness by the task force.

### Feedback reports motivated action planning

The feedback reports informed hospital providers about their acute stroke treatment times and rates. Awareness of deficiencies in their performance led to hospital staff and leadership meetings to devise strategies for improvement. Internal motivation for development of these strategies came from goal setting, teamwork, and support from leadership. The feedback reports served as a reminder to ED staff of the goal of decreasing stroke treatment times, motivating them to find ways to achieve that goal. As stated by one ED staff member about reasons for perceived faster stroke treatment times, *“...partly I think is like the feedback that we’ve been doing and getting from you guys at U of M and then also like going over, like I present like we had 4 thrombolytics [tPA] administrations and these were our times and there were the delays and these were the reasons for the delays. Just so like everyone knows that it’s like something that’s being measured and it’s somewhat of a like…it needs to be a priority, and you have to move things and you have to move fast.”* Meetings were also a forum to reinforce the goal of improving stroke treatment times and to foster the spirit of teamwork, as exemplified by the following quote:

*“I think all the way from our ED operations meeting down to our management meeting to the ED nursing, I think we all knew the objective and goal right from the get-go from our stroke meetings. And I think knowing the vision, the strategic plan to it, I think we all just kinda jumped on board.”*

Hospital leadership and senior ED clinical leadership worked together with ED providers to support implementation strategies, as the following quote illustrates from a senior nurse manager regarding senior ED clinical providers’ support of acute stroke protocols “*I think there’s a lot of support from, you know, senior leaders all the way down that this is something huge, something that’s right for our patients, and I believe the physicians feel very supported.”*

### How feedback reports led to implementation strategies

The five implementation strategies that hospital staff generated independently of the research team arose out of the feedback reports as follows:

The feedback reports stimulated the feedback recipients to assess whether the goal of feedback was achievable and within their control. Specifically, hospital staff evaluated whether the goal of increasing the amount and speed of tPA delivery could be achieved. Hospital staff realized that they needed additional training in how to deliver tPA and access to tPA in the ED. According to one ED staff member about their need for training on giving tPA, *“not everyone I don’t think was very well educated in tPA and the dosing and everything…. So every year, we have to show that every staff member knows how to mix the tPA, set it up on the [intravenous] pump.”* One ED staff member commented about their lack of familiarity with how to access tPA *“like the first stroke we had, it’s like, oh, where do we get the tPA? Do we wait for that to come from Pharmacy, what are we doing,....”*

The feedback reports stimulated hospital staff to reflect upon their current stroke processes and compare their current processes to other institutions, which served as an external source of motivation for action planning. They learned of other institutions’ protocols during EMS quality improvement meetings attended by representatives of each hospital in the county. According to one senior nurse manager*, “so we knew [local hospital x] was doing it [creating stroke protocols]. And then [local hospital y] was getting ready…well, because they all sit on the [EMS quality improvement council].”* Further comments from the senior nurse manager explains their motivation to develop stroke protocols, *“so I think the big driver, honestly, looking back when we started piloting the program was frankly [2 local hospitals] are setting up these protocols, and we’re gonna be left behind if…we’re not gonna keep up with the community standards if we don’t do something similar, and then I think ultimately it just makes sense.”* They identified that there was a need to create a protocol for acute stroke care. “*So, like when it first started or even before then, it was, oh, we have a stroke. Well, what…nobody knew what do you do with a stroke.”* They also obtained ideas for organizational change from the protocols of other institutions as this quote from an ED clinical leader illustrates: *“So, in looking in other peoples’ data, it seems that it made more sense to get them [stroke patients] directly to CT so that the timing is, you know, much shorter.”*

#### Implementation strategies

We identified five implementation strategies that were implemented by hospital staff after initiation of A&F: stroke protocols, stroke care education and tPA competency training, timing of head computed tomography (CT), increased access to tPA in the emergency department, and support for tPA delivery. The implementation strategies were focused on raising awareness around the time-sensitive nature of stroke treatment as well as implementing guideline-concordant stroke treatment. The implementation strategies are named, defined, and specified in Table 1. All implementation strategies were directed towards ED providers and the organization. We describe each individually:

##### Strategy 1: detailed stroke care protocol folders located in the Emergency Department

Stroke administrative staff and clinical leaders created folders with detailed step-by-step protocols for delivering acute stroke care in the ED. These folders were placed in the ED with other protocols in a high-volume common area to increase accessibility.

The purpose of this implementation strategy was for staff to use the folder as a detailed, step-by-step guide/checklist of where to find tPA and how to administer it. We hypothesize that providing detailed stroke care protocol folders in the ED used the mechanism of enablement by enhancing provider psychological capability to think through the steps necessary to administer tPA, which led to increased use of the protocol folders and resulted in the outcome of guideline-concordant tPA use.

**Fig. 2:**
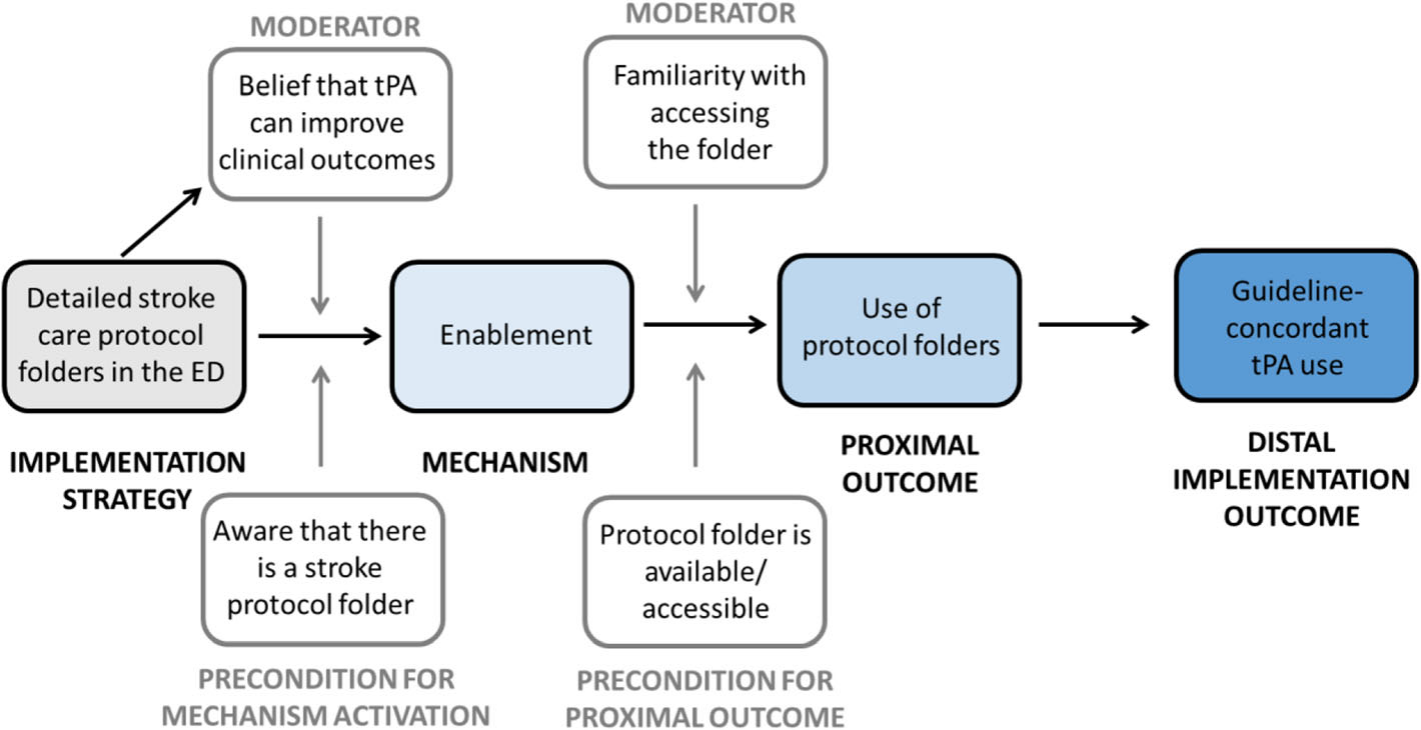
Causal pathway model for implementation strategy of detailed stroke care protocol folders

The causal pathway model for the implementation strategy of detailed stroke care protocol folders is depicted in Fig. 2.

According to one ED staff member about the stroke protocol, *“When you’re educating, training somebody, you’re like, oh, so here’s our list of protocols, our list of, you know, the different folders that we have… We have one for the [acute stroke care protocol], which is our stroke,..… So everybody is aware that there is information there to get even if you’re not the one that has the information.”*

Evidence supporting that stroke care protocol folders increased psychological capability is exemplified by the following quote: *“Like there’s a book that we go to when we need to find out, you know, what needs to be done. So, that’s works so it’s not everybody running around going, hey, do you remember how to do this, do you remember how to do this.”*

##### Strategy 2: stroke care education and tPA competency training

One of the ED quarterly competency training sessions was devoted to stroke care protocols and tPA administration. ED nurses and providers were trained on how to implement the acute stroke care protocol. ED nurses were educated on tPA dosage administration based on body weight and how to program the intravenous pump. ED nurses were instructed on when and where during acute stroke care to retrieve tPA and patient monitoring after tPA administration. Information disseminated during annual training was reinforced during daily 6-min staff ‘huddles’/ meetings at the beginning of each ED shift.

We hypothesize that through training on stroke care and tPA, ED providers increased their knowledge and self-efficacy in providing guideline-concordant tPA treatment.

**Fig. 3:**
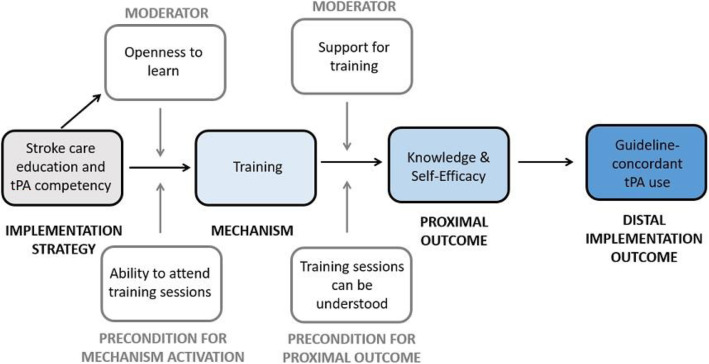
Causal pathway model for implementation strategy of stroke care education and tPA competency

The causal pathway model for the implementation strategy of stroke care education and tPA competency is depicted in Fig. 3.

One ED staff member stated, “*Quarterly competency. We were just trying to show like... Joint Commission or CMS [Centers for Medicare and Medicaid Services] and improve staff knowledge.... and comfortability that we were gonna do quarterly competencies.…So part of the quarter competency that I added for the ER was doing the tPA and stroke.”*

With respect to the influence of training, one staff member stated “*I remember when I first started, when you had to give tPA, it was like a huge, scary thing, so being more familiar with it has definitely made you more comfortable giving it I guess.”*

##### Strategy 3: modification of the timing of computed tomography (CT) brain imaging in acute stroke care

A protocol was put in place to take stroke patients directly from the ambulance to the CT scanner upon hospital arrival. ED providers were more consistently notified by emergency medical services (EMS) about stroke patients en route to the hospital. An acute stroke notification was then sent to all hospital personnel involved in stroke care, informing that a stroke patient was en route. The CT scanner would then be available for the stroke patient upon his/her arrival. As a CT scan must be obtained and reviewed before administering tPA, this protocol aimed to increase the speed of tPA delivery.

We hypothesize that taking the patient directly to the CT scanner upon arrival to the ED was a new protocol that accomplished the function of environmental restructuring, reducing the time to obtain CT imaging and increasing the timeliness of tPA administration.

**Fig. 4:**
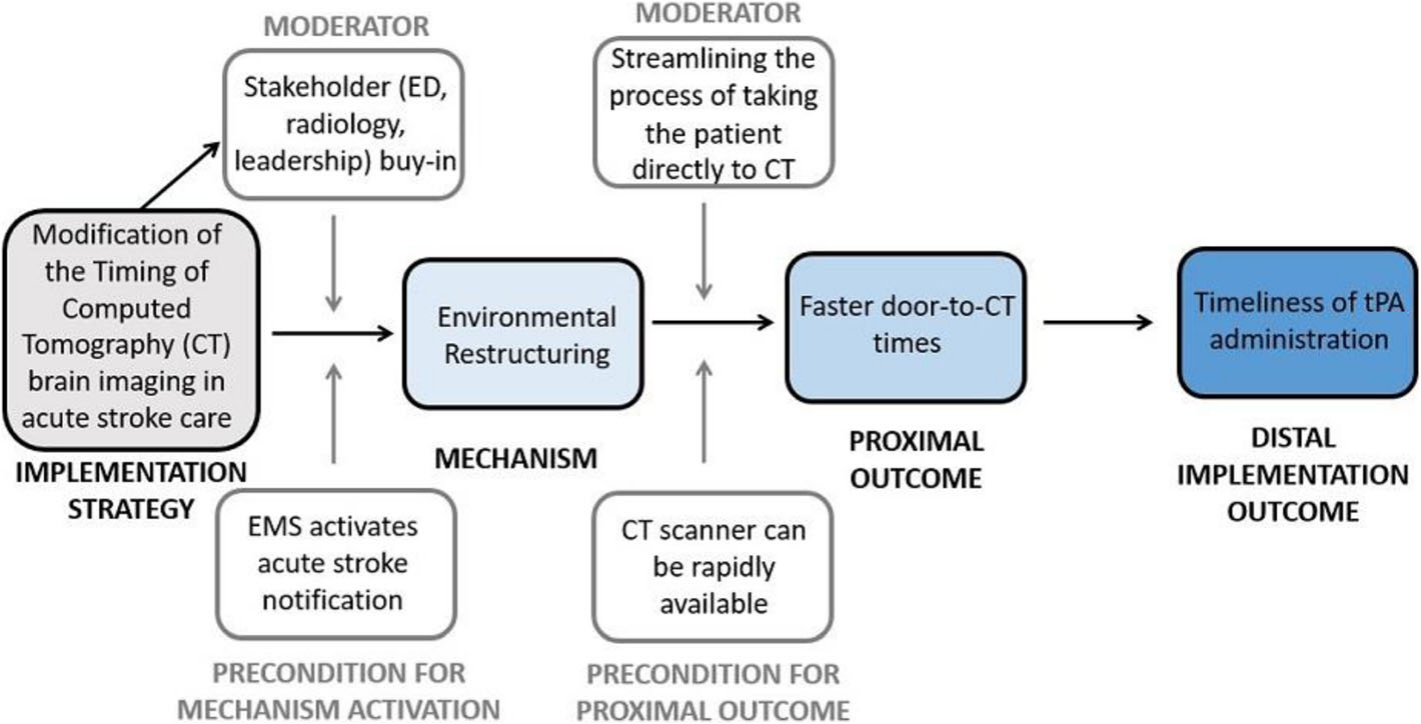
Causal pathway model for implementation strategy of modification of the timing of CT brain imaging in acute stroke care

The causal pathway model for the implementation strategy of modification of the timing of CT brain imaging in acute stroke care is depicted in Fig. 4.

According to an ED provider, *“Well, we implemented the [pre-arrival acute stroke notification], so we can activate within the department so that we would get CT scan ready and the physicians know what’s coming in. That’s been a big change. I think it really has helped our times from door to CT [from patient arrival to obtaining CT scan] and door to tPA [from patient arrival to administering tPA] and any further intervention that’s needed.”*

The following quotes from 2 senior nurse managers exemplifies that the feedback report on their tPA treatment times motivated the new direct to CT scanner implementation strategy, *“Before they would come in by EMS, they would go into a room, and then the nurse would start to triage the patient. Sometimes they would do the entire triage before a physician would be notified, you know, and it just muddied up the process”* and *“We just noticed that that was a lot of our holdup in our tPA times because we have all the data, and getting to CT was always a problem.”*

##### Strategy 4: increased access to tPA in emergency department

Tissue plasminogen activator, which had previously been only available at the ED pharmacy, was stored in automated medication-dispensing cabinets in the ED; it has to be reconstituted with sterile water prior to administration. Storing tPA in the ED increased ED nurses’ access to the medication and eliminated delay in waiting for pharmacy to deliver the medication.

We believe that storing tPA in the ED was a strategy that accomplished the function of environmental restructuring, thereby enhancing the efficiency with which tPA could be accessed and the timeliness of tPA administration.

**Fig. 5:**
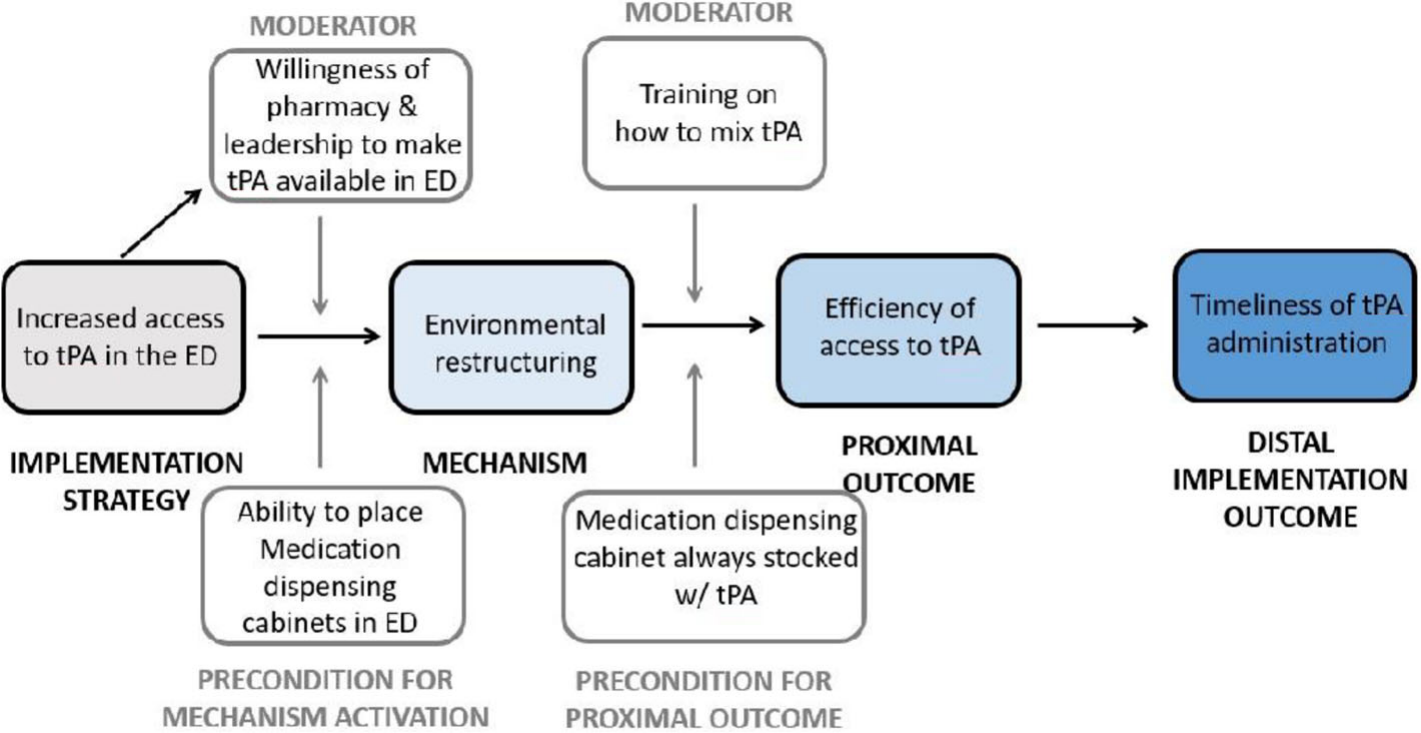
Causal pathway model for Implementation strategy of increased access to tPA in emergency department

The causal pathway model for the implementation strategy of modification of the timing of increased access to tPA in emergency department is depicted in Fig. 5.

One ED provider stated, *“There’s also access to the tPA, which is much easier now than it was before. There’s one in each AcuDose [medication dispensing cabinet] in each of the pods, so we’re able to get that, and I think that there’s more education so everybody’s kind of on the same page as far as what we’re doing and when we’re supposed to be doing it.”*

##### Strategy 5: staff-supported implementation of acute stroke care protocols

When an acute stroke patient is en route to the ED, an acute stroke notification is activated. Experienced nurses and physicians assist the ED team responsible for the acute stroke patient as they carry out the acute stroke care protocol, providing assistance or verbal reminders about the protocol when needed.

We hypothesize that support of the acute stroke care protocol increased the speed and efficiency of protocol execution through enablement, that is enhancing the psychological capability of staff to carry out the protocol. This strategy increased the timeliness of tPA administration.

**Fig. 6:**
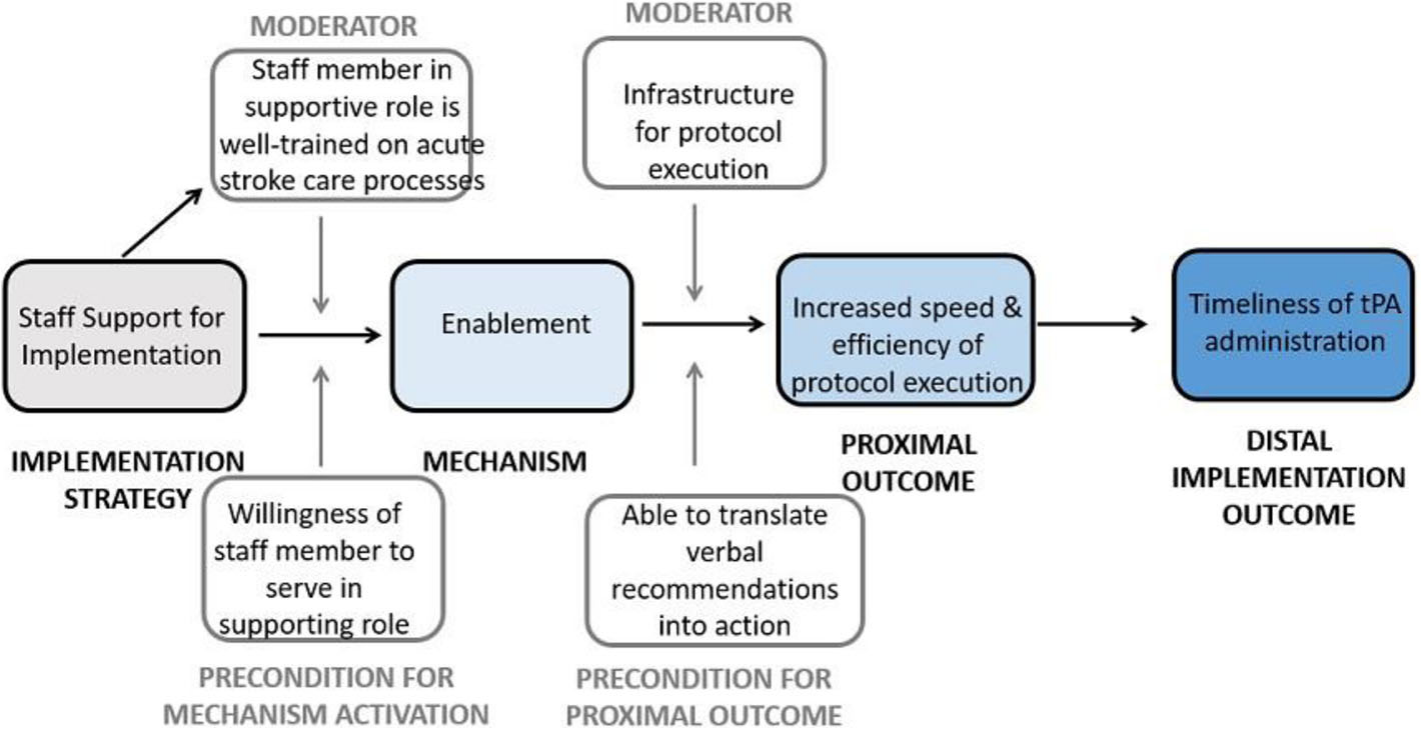
Causal pathway model for implementation strategy of staff-supported implementation of acute stroke care protocols

The causal pathway model for the implementation strategy of staff-supported implementation of acute stroke care protocols is depicted in Fig. 6.

According to one ED provider, *“You’re going to see a bunch of staff members you know go and help, and you know if there’s new people that are having to administer tPA, senior staff members are right by their side and walking them through it.”*

The following quote from an ED staff member exemplifies how staff support for implementation increases psychological capability which leads to efficiency of execution of the protocol for administering tPA: *“so everybody kind of knows what needs to be done, and they plug themselves in wherever's needed. There's no like, you do this, you do this, no one's kind of directing. Everybody just knows. All the senior nurses know what needs to be done and they fill the holes.”*

### Perceived outcomes

Overwhelmingly, the participants reported that the implementation strategies improved patient care. For example, in regards to the stroke care protocol, one nurse reported, *“Well, it's [tPA protocol packets] working. And when something works, people will take it more serious. I don’t know if that's the right way to say, but you know the system is working so then you stick with it.”* Another nurse similarly reported about the stroke implementation strategies, *“A lot of times we're given systems that don’t really work. Then we try this, and it doesn’t work that well. Well, if something works, then you know it makes our job easier, it's better for the patient, it'll usually stick.”* This culminated in the perception of improved care, as one ED provider noted, *“It seems like we're getting them [stroke patients] treated more quickly.”* One senior nurse manager uses data provided in the feedback report as evidence that implementation strategies are resulting in faster stroke treatment times in stating *“So, I think some of the changes that we’ve seen are system improvements with regards to the delivery of acute care and thrombolytics, and I think the direct EMS to CT is then helpful, and certainly the data [from the feedback reports] would reflect it’s been helpful in decreasing our times to thrombolytic administration for patients that come in through that route.”*

The study was underpowered to detect a change in the primary outcome, namely rate of tPA use. After completion of the research team-driven feedback report period, the hospital independently continued creating and distributing monthly feedback reports.

## Discussion

We have demonstrated the process by which hospital staff initially selected A&F as an implementation strategy to optimize acute stroke care processes, stimulated by a determinant assessment conducted by the research team. Importantly, we have also shown that this initial strategy facilitated new implementation strategies. Our results provide insight into the mechanism of action of A&F reports. Our qualitative approach made it possible to identify these actions.

A&F is an effective means of improving implementation of evidence-based practices [17], but there is variability in the effectiveness of A&F which might partially be explained by a lack of understanding of how it works [18]. Our study helped to address this shortcoming. Qualitative methods have been used to identify why A&F can fail to elicit a behavioral response [19] or to describe the process of action planning [20]. Our A&F tool provided feedback to the local hospital about their acute stroke care performance using national standards as the benchmark. We emphasize the importance of this approach as feedback based on national A&F tools have been shown to be difficult to interpret and apply at the local hospital level [21–23]. Our study is unique in its identification of *how* A&F promotes further implementation of evidence-based practices through specific processes that caused hospital staff to generate new strategies, independent of the research team.

The key question, then, becomes why did A&F lead to multiple other interventions in this setting? One explanation is that this commonly occurs, but is not explored after the implementation of A&F. Another possibility is that A&F sparked internal and external sources of motivation. The A&F process stimulated internal sources of motivation such as teamwork and support from leadership. Goal setting was the initial step to unify hospital staff towards a common purpose. Once the goal of improving stroke treatment rates and times was defined, hospital staff and leadership worked as a team to achieve the goal. One may argue that the goal of increasing stroke treatment rates and decreasing stroke treatment times lacked specificity, as neither the research team nor hospital staff defined quantitatively by how much tPA rates should increase or treatment times should decrease. However, the goal was more specific than ‘to improve’ or ‘do better’. Rather, we used national standards of tPA treatment as the benchmark for performance standards. The national guideline of treating stroke patients within 60 minutes of hospital arrival was displayed on each feedback report. Consistent with the goal-setting theory of Locke and Latham [24], hospital staff were committed to achieving the goal because they viewed the goal as important and attainable if provided with the necessary supports. Staff felt supported by hospital leadership who were motivated to understand and improve their performance and provided the resources needed to make organizational-level changes. This collaboration between leadership and their staff has been recognized as a successful approach for promoting the generation of strategies to achieve a goal [24]. Working as a team with the support of hospital leadership, ED staff recognized that the implementation strategies improved stroke care.

In addition to internal sources of motivation, hospital leadership was driven by external sources of motivation for improving acute stroke care. Hospital leadership compared their acute stroke care processes with other hospitals in the region. While the feedback report did not include comparative data, hospital leadership learned of other hospitals’ stroke processes during regional meetings. We propose that the feedback reports increased leadership’s focus on acute stroke processes, thereby causing them to attend to and learn from the practices that other clinical sites employ to meet standards of stroke care. Other research has similarly found that comparison of a clinical site’s performance to average peer performance can bridge the information–intention gap, that is bridge the gap between feedback and intention to change [25].

Given the success of A&F in stimulating change at the organization level, the hospital continues to use A&F for quality assurance; our approach to A&F, which allows them to continue to generate the feedback reports, enables them to continue the intervention. Consistent with CP-FIT theory [4], the feedback process, contextual factors, and characteristics of the recipients of feedback likely all contributed to the success of the initial and ongoing A&F intervention. Specifically, the emergency department providers worked in teams towards a common goal of improving acute stroke care with hospital leadership and staff members serving as champions supporting implementation efforts, motivated by the information they received about their performance through the feedback reports. The ability of A&F to lead to clinical performance improvement depends on behavior change and also the motivation and cooperation of key stakeholders in the feedback process and their ability to act beyond the initial research team-driven strategy.

Our study suffers from some important limitations. First, the study setting was a single hospital in one community. As a result, we have no contextual variation to allow us to study whether other hospitals would take on the relatively independent action that providers took on at this hospital. Literature suggests that this is a relatively rare event [21–23]. Furthermore, we do not know how much A&F led to the hospital’s generation of implementation strategies over contextual factors such as the need to maintain hospital stroke accreditation and the desire to meet the same standards of care as other local hospitals. We also acknowledge that we do not know whether the hospital implemented the strategies for every acute stroke patient and the degree to which the strategies were in development prior to the A&F report period. Moreover, the study was underpowered to detect a change in the primary outcome, namely rate of tPA use. In addition, our findings might not be generalizable to hospitals in resource-rich communities that may have different barriers to optimizing acute stroke care. Finally, our causal pathway models postulate how implementation strategies produce their effects based on the analysis of our qualitative data. We acknowledge that there may be different approaches to defining mechanisms of action [26]. We used synthesized member checking to confirm that participants agreed with the implementation strategies described, but did not confirm that they agreed with the proximal outcomes, distal outcomes, preconditions, and moderators in the causal pathway models. Future studies could test the hypothesized pathways.

## Conclusions

In summary, we have demonstrated how A&F was used and amplified, leading to additional implementation strategies, by a hospital to improve acute stroke care. Rooting our A&F strategy in CP-FIT theory allowed us to use the tenets of the theory to identify how A&F led to the development of different implementation strategies. Feedback reports clearly stated the implementation goal of increasing tPA delivery and decreasing tPA treatment times. Hospital staff used internal and external sources of motivation to remain engaged in the process of developing implementation strategies to achieve the goal. Different implementation strategies arose as A&F stimulated the hospital team to reflect on current practices and the achievability of the goal. The research team remained present and supportive throughout the hospital team’s action planning and provided them with the tools needed to independently sustain A&F after the completion of the research. Future research will evaluate whether a theory-based A&F strategy can similarly support other clinical sites to develop strategies to improve their acute stroke treatment processes.

## Supplementary Information

**Additional file 1.** Interview guide for determinant assessment.

**Additional file 2.** Worksheet for scoring implementation strategies by APEASE criteria.

**Additional file 3.** Description of development of the A&F report.

**Additional file 4.** Sample feedback report.

**Additional file 5.** Sample interview guide.

**Additional file 6.** Standards for Reporting Qualitative Research checklist.

## Data Availability

The datasets used and/or analyzed during the current study are available from the corresponding author on reasonable request.

## Abbreviations

A&F: Audit and feedback
APEASE: Affordable, practical, effective, acceptable, safe, and equitable
COM-B: Capability, opportunity, motivation, and behavior
CP-FIT: Clinical Performance Feedback Intervention Theory
CT: Computed tomography
ED: Emergency department
EMS: Emergency medical services
ER: Emergency room
FDA: Food and Drug Administration
TDF: Theoretical Domain Framework
TICD: Tailored Implementation for Chronic Disease
tPA: Tissue plasminogen activator
