# Supplementary material for: A step toward understanding the mechanism of action of audit and feedback: a qualitative study of implementation strategies
Source: Implement Sci. 2021 Apr 1;16:35. doi: 10.1186/s13012-021-01102-6 (PMC8017642; doi:10.1186/s13012-021-01102-6)
Supplement: Supplementary file 2 — Additional file 2. Worksheet for scoring implementation strategies by APEASE criteria. [file 13012_2021_1102_MOESM2_ESM.docx]

**Optimizing Acute Stroke Treatment: Ranking Interventions on the APEASE Criteria**

Please rank each of the interventions based on each criterion below with 1 having the least desired score and 5 having the most desired score.

|  | Feedback Reports | Restructured Consent Process | Acute Stroke Team | Stroke Champion | Documentation of LKN |
| --- | --- | --- | --- | --- | --- |
| **Affordability** | **The intervention is within an acceptable budget in which it can adequately be delivered.** | | | | |
| 1 – Strongly Disagree  2 – Disagree  3 – Not sure  4 – Agree  5 – Strongly Agree |  |  |  |  |  |
| **Practicability** | **The intervention can be delivered as designed to the target population.** | | | | |
| 1 – Strongly Disagree  2 – Disagree  3 – Not sure  4 – Agree  5 – Strongly Agree |  |  |  |  |  |
| **Effectiveness** | **The effect size of the intervention in relation to the desired objectives in a real world context is ideal.** | | | | |
| 1 – Strongly Disagree  2 – Disagree  3 – Not sure  4 – Agree  5 – Strongly Agree |  |  |  |  |  |
| **Acceptability** | **The intervention is appropriate for all stakeholders (patients, ER techs, nurses, physicians, administrators).** | | | | |
| 1 – Strongly Disagree  2 – Disagree  3 – Not sure  4 – Agree  5 – Strongly Agree |  |  |  |  |  |
| **Side-Effects/ Safety** | **The intervention has the fewest possible unintended consequences.** | | | | |
| 1 – Strongly Disagree  2 – Disagree  3 – Not sure  4 – Agree  5 – Strongly Agree |  |  |  |  |  |
| **Equity** | **The intervention may reduce disparities between different groups (age, sex, race, gender).** | | | | |
| 1 – Strongly Disagree  2 – Disagree  3 – Not sure  4 – Agree  5 – Strongly Agree |  |  |  |  |  |
| **TOTAL** |  |  |  |  |  |
