# Supplementary material for: A step toward understanding the mechanism of action of audit and feedback: a qualitative study of implementation strategies
Source: Implement Sci. 2021 Apr 1;16:35. doi: 10.1186/s13012-021-01102-6 (PMC8017642; doi:10.1186/s13012-021-01102-6)
Supplement: Supplementary file 3 — Additional file 3. Description of development of the A&F report. [file 13012_2021_1102_MOESM3_ESM.docx]

**Acute stroke treatment reporting tool**

We used an iterative, user-centered design approach to develop a reporting tool^1^. A key requirement for the design of the tool was that the ED team could use it independently of the research team to produce visual displays for feedback reports and presentations. We developed the acute stroke treatment reporting tool for ED teams as a Javascript-based web application that generates a single image to communicate about the timing of stroke treatment using 4 performance measures (Figure 1). The image can be pasted into reports or presentations for teams to view and interpret. The tool is implemented in a web application that accepts patient-level event data, but does not transmit patient data, processing data only locally in the web-browser (Figure 2). The tool is freely available for use at acutestrokereports.org. The software for the tool is available under an open source license at <https://github.com/Display-Lab/stone-throw>. The tool is likely to be scalable across hospital ED settings because of its minimal requirements and the ability of the visual display it produces to be interpretable by clinicians, and to be pasted into any report document or presentation.

**Use of the acute stroke treatment reporting tool**

Since December 2018, feedback report distribution was transitioned to the hospital Stroke Coordinator, who has successfully continued creating and distributing monthly feedback reports independently. The feedback report appears to have been an important component of the program to implement acute stroke by enabling the clinical team to maintain awareness about the quality of care and to monitor improvement over time as organizational and team level changes were implemented to improve acute treatment of stroke.

**Figure 1.** Image generated by the reporting tool for use in feedback reports and presentations.

**Figures shown are not actual hospital data and were created for this sample report.**

**
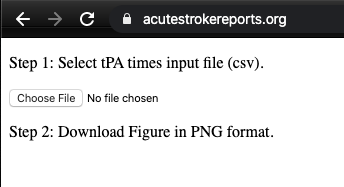
**

**Figure 2.** Browser-based user interface for the acute stroke treatment reporting tool.

Reference

1. Landis-Lewis Z, Kononowech J, Scott WJ, et al. Designing clinical practice feedback reports: Three steps illustrated in Veterans Health Affairs long-term care facilities and programs. *Implement Sci*. 2020;15(1). doi:10.1186/s13012-019-0950-y
