# Supplementary material for: A step toward understanding the mechanism of action of audit and feedback: a qualitative study of implementation strategies
Source: Implement Sci. 2021 Apr 1;16:35. doi: 10.1186/s13012-021-01102-6 (PMC8017642; doi:10.1186/s13012-021-01102-6)
Supplement: Supplementary file 4 — Additional file 4. Sample feedback report. [file 13012_2021_1102_MOESM4_ESM.docx]

**Stroke Feedback Report**

***The current guideline recommends that healthcare professionals treat eligible stroke patients with tPA within 60 minutes of arrival to the hospital.***

- Tissue plasminogen activator (tPA) is the only approved acute stroke treatment. tPA greatly increases the chance that a stroke patient will have a good outcome.
- tPA works better the earlier it is given.
- Major medical organizations including the American Heart Association and American College of Emergency Physicians support treating eligible stroke patients with tPA.
- If you have any questions please contact the Stroke Program Coordinator.

**Figures shown are not actual hospital data and were created for this sample report.**

**GRAPH 1: PERCENTAGE OF ISCHEMIC STROKE PATIENTS TREATED WITH TPA**

**
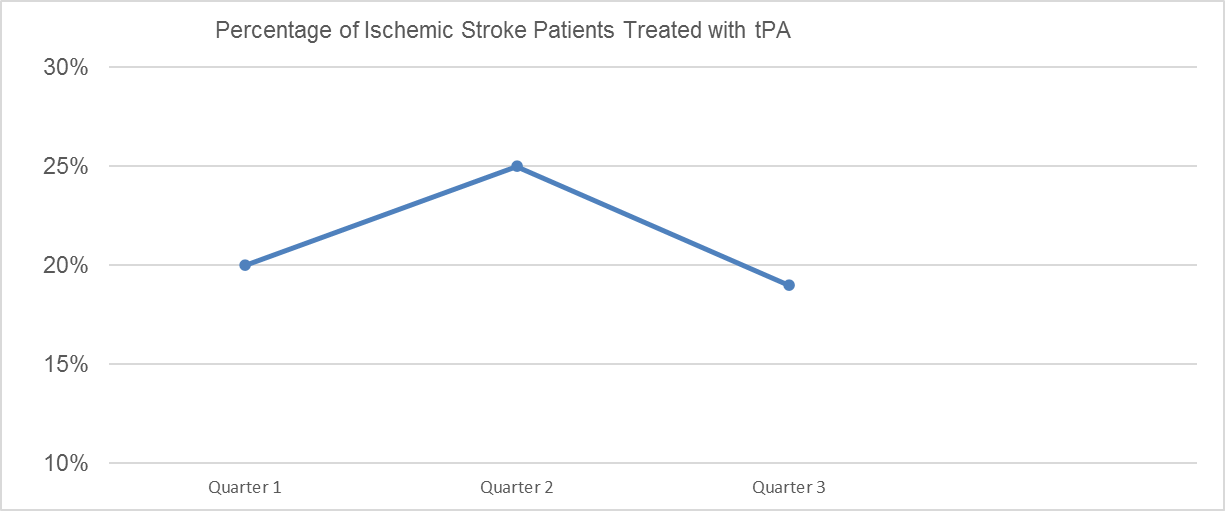
**

- Time is measured in quarters on the horizontal line

**Figures shown are not actual hospital data and were created for this sample report.**

**GRAPH 2: ACUTE STROKE TREATMENT TIMES: SHORTER TIMES ARE BETTER**

**Figure 1**

**Figure 3**

**Figure 2**

Figure 1: Door to Treatment shows the overall time from when the patient arrived to the hospital (Door) to when the patient received tPA treatment.

Figure 2: Door to Doctor shows the time from when the patient arrived to the hospital (Door) to when the patient first saw a doctor.

Figure 3: Door to Head CT shows the time from when the patient arrived to the hospital (Door) to when the patient received a head CT scan.

**Figures shown are not actual hospital data and were created for this sample report.**
