# Supplementary material for: A step toward understanding the mechanism of action of audit and feedback: a qualitative study of implementation strategies
Source: Implement Sci. 2021 Apr 1;16:35. doi: 10.1186/s13012-021-01102-6 (PMC8017642; doi:10.1186/s13012-021-01102-6)
Supplement: Supplementary file 5 — Additional file 5. Sample interview guide. [file 13012_2021_1102_MOESM5_ESM.docx]

*Hello I am [your name] from the Neurology Department at the University of Michigan Health System. We have partnered with [hospital name] to work together to improve acute stroke care. Before we begin I want to let you know that we are interested in your comfort in this interview and we are here to talk about anything you would like to talk about.*

*Since October the Stroke Program has been providing feedback reports about acute stroke treatment. The reports include the proportion of stroke patients who received tPA, treatment times and the documentation of last known normal. The purpose of this interview is to learn more about how the feedback reports are used and your thoughts about how changes have been made to acute stroke care.*

I am going to start with a few basic questions---

1. **Which unit do you work on**? (Check **ONE** if you only work on one unit. If you work on more than one unit—i.e., in the ED and neurology floor, then check **ALL** that apply):
   1. _____Emergency Department
   2. _____Neurology Floor
   3. _____Other (Please specify below):
2. **What is your position title**? (Check **ONE**; if you have more than one position, check the one that you **work most often**):
   1. _____Attending Physician
   2. _____Resident Physician
   3. _____Physician Assistant
   4. ­­­_____Nurse Practitioner
   5. _____Nurse
   6. _____Emergency Department Technician/Medical Assistant
   7. _____Other (Please specify below):

**Reflect back on the last 6 months regarding acute stroke patient care in the emergency department.**

1. **What changes in acute stroke care have you noticed at [hospital name]?^1^**
   1. Have you noticed changes in the social environment at [hospital name]?
      1. If so, what changes?
   2. Have you noticed changes in the physical or structural environment?
      1. If so, what changes?
   3. What do you believe influenced/led to these changes?
2. **What role has the senior leadership/clinical management played in the acute stroke care?**
   1. What is their management style? Is innovation rewarded?
   2. How open are they to changes in acute stroke care?
   3. How is stroke prioritized compared to other disease processes in ED?
   4. Are clear goals set?
   5. Are the necessary resources available to support improvements?
   6. What is the role of clinical champions?
3. **We are particularly interested in the improvements in door to treatment times that have occurred over the last 6 months.**
4. What do you think led to these changes?
5. What role did the feedback report play in this, if any?
   - 1. (If interviewee indicates an endorsing answer), what about the feedback report may have made a difference?

**In theory, we assume that providing providers with feedback will promote changes in patient care**

1. **Think about the most recent time you saw the acute stroke feedback report.**
   1. Tell me about when you last recall viewing the feedback report.
   2. Where did you review the information from the feedback report? (E-mail, staff area, etc.)
   3. Before receiving the feedback reports, were you aware of how [hospital name] was performing in treatment of acute stroke patients?
   4. Who did you discuss it with and why did you decide to discuss with these people?
      1. Were these feedback reports discussed in a formal meeting setting? If yes, in which meetings?
   5. Do you feel that these reports gave you an accurate view of [hospital name’s] performance?
   6. Did you find the reports to be engaging?
   7. What was helpful? What was not helpful?
   8. What additional information in the feedback report do you believe would help with continuing improvement?
2. **How did reviewing the acute stroke feedback report influence your patient-care?**
   1. If interviewee made changes,
      1. What did you think motivated you to make these changes?
   2. If interviewee made no changes,
      1. Do you feel that any changes are necessary?
3. **Have any specific people or groups of people been influenced by the feedback reports?**
4. If so, who do you think may have been influenced by the feedback reports? Clinical leader? Opinion leader? Stroke champion?
5. In what ways do you believe they were influenced?
6. Do you believe they were influential to others? How so?
7. Did they seem to have more motivation, awareness, or a better understanding because of the feedback report?
8. Who do you believe has been the least impacted by the feedback report?
9. **What role do you think the feedback reports can have in sustaining changes that have been implemented, if any?**
10. **In your opinion, if the feedback reports were to be generated at another hospital in the future, how often would you recommend the feedback reports to be given (Quarterly, monthly)?** **(Prompt: How often is too often? How often is not often enough?)**
    1. Do you believe that monthly or quarterly is better?
    2. When do you believe the feedback reports should stop being given? (Prompt: Is the length of time dependent on reaching a specific goal? Should they be continued indefinitely? Should they only be given when a goal hasn’t been reached?)
11. **Is there anything else you would like to say or that you think it would be important for me to know?**

Thanks so much!

1. Boyce MB, Browne JP, Greenhalgh J. Surgeon’s experiences of receiving peer benchmarked feedback using patient-reported outcome measures: a qualitative study. Implementation Science. 2014;9(1):84.

2. Michie S, Johnston M. Theories and techniques of behaviour change: Developing a cumulative science of behaviour change. Health Psychology Review. 2012;6(1):1-6.

3. Stephan A-J, Kovacs E, Phillips A, Schelling J, Ulrich SM, Grill E. Barriers and facilitators for the management of vertigo: a qualitative study with primary care providers. Implementation Science. 2018;13(1):25.
